# Assessment of Intervertebral Lumbar Disk Herniation: Accuracy of Dual-Energy CT Compared to MRI

**DOI:** 10.3390/jcm14197000

**Published:** 2025-10-03

**Authors:** Giuseppe Ocello, Gianluca Tripodi, Flavio Spoto, Leonardo Monterubbiano, Gerardo Serra, Giorgio Merci, Giovanni Foti

**Affiliations:** 1 Department of radiology, Messina University Hospital, 98124 Messina, Italy; giuseppeocello27@gmail.com (G.O.); gianlucatripodi.gt@gmail.com (G.T.); 2 Department of radiology, IRCCS Sacro Cuore Don Calabria Hospital, 37024 Negrar, Italy; flaviospoto@hotmail.it (F.S.); leonardo.monterubbiano@sacrocuore.it (L.M.); 3 Department of Anesthesia and Analgesic Therapy, IRCCS Sacro Cuore Don Calabria Hospital, 37024 Negrar, Italy; gerardo.serra@sacrocuore.it (G.S.); giorgio.merci@sacrocuore.it (G.M.)

**Keywords:** disk hernia, dual energy CT, MRI, accuracy, computed tomography

## Abstract

**Background:** Lumbar disk herniation is a common cause of low back pain and radiculopathy, significantly impacting patients’ life quality and functional capacity. Magnetic Resonance Imaging (MRI) remains the gold standard for its assessment due to its superior soft tissue contrast and multiplanar imaging capabilities. However, recent advances in spectral computed tomography (CT), particularly dual-energy CT (DECT), have introduced new diagnostic opportunities, offering improved soft tissue characterization. **Objective:** To evaluate the diagnostic performance of DECT in detecting and grading lumbar disk herniations using dedicated color-coded fat maps. **Materials and Methods:** A total of 205 intervertebral levels from 41 consecutive patients with lumbar symptoms were prospectively analyzed. All patients underwent both DECT and MRI within 3 days. Three radiologists with varying years of experience independently assessed DECT images using color-coded reconstructions. A five-point grading score was attributed to each lumbar level: 1 = normal disk, 2 = bulging/protrusion, 3 = focal herniation, 4 = extruded herniation, and 5 = migrated fragment. The statistical analysis included Pearson’s correlation for score consistency, Cohen’s Kappa for interobserver agreement, generalized estimating equations for a cluster-robust analysis, and an ROC curve analysis. The DECT diagnostic accuracy was assessed in a dichotomized model (grades 1–2 = no herniation; 3–5 = herniation), using MRI as reference. **Results:** A strong correlation was observed between DECT and MRI scores across all readers (mean Pearson’s r = 0.826, *p* < 0.001). The average exact agreement between DECT and MRI was 79.4%, with the highest concordance at L1–L2 (86.7%) and L5–S1 (80.4%). The interobserver agreement was substantial (mean Cohen’s κ = 0.765), with a near-perfect agreement between the two most experienced readers (κ = 0.822). The intraclass correlation coefficient was 0.906 (95% CI: 0.893–0.918). The ROC analysis showed excellent performance (AUC range: 0.953–0.986). In the dichotomous model, DECT demonstrated a markedly higher sensitivity than conventional CT (95.1% vs. 57.2%), with a comparable specificity (DECT: 99.0%; CT: 96.5%) and improved overall accuracy (98.4% vs. 90.0%). Subgroup analyses by age and disk location revealed no statistically significant differences. **Conclusions:** The use of DECT dedicated color-coded fat map reconstructions showed high diagnostic performance in the assessment of lumbar disk herniations compared to MRI. These findings support the development of dedicated post-processing tools, facilitating the broader clinical adoption of spectral CT, especially in cases where MRI is contraindicated or less accessible.

## 1. Introduction

Lumbar intervertebral disk herniation is one of the leading causes of low back and radicular pain worldwide, with an estimated prevalence between 1% and 5% in the general adult population [1,2,3]. It most commonly affects individuals aged 30 to 50, with a slight male predominance. At least 50% of the population is expected to experience an episode of low back pain or sciatica during their lifetime. Disk herniation results from the degeneration of the intervertebral disk, composed of a central nucleus pulposus and a peripheral annulus fibrosus. Degenerative changes lead to dehydration in the nucleus pulposus, predisposing the disk to annular fissures [4,5]. Nuclear material may migrate, resulting in disk protrusion or extrusion into the spinal canal and potentially compressing or inflaming adjacent nerve roots. These processes may cause a clinical spectrum ranging from localized back pain to complex neurological deficits [6]. MRI allows for the precise visualization of the disk herniation (protrusion, extrusion, sequestration) and its relationship to neural structures [7]. Additionally, MRI permits the grading of the herniation based on the location (central, subarticular, foraminal, extraforaminal) and the degree of neural or canal compromise [8]. Dual-energy CT (DECT) has seen a growing application in musculoskeletal imaging [9,10], offering a high-resolution and soft tissue contrast, combined with bone reconstruction algorithms. In spinal imaging, DECT has been proved to be an accurate imaging tool for diagnosing several pathological conditions, including vertebral fractures and spondylodiscitis [11,12,13]. Also, DECT can be used for the reduction in artifacts due to metal hardware, for the assessment of surgically treated spines [14,15,16], and for the assessment of lumbar foraminal stenosis [17]. In addition to this, DECT has been used for the evaluation of disk herniation in lumbar spines [18,19]. In the paper of Booz et al. [20], color-coded virtual noncalcium reconstructions showed a 91% sensitivity and 92% specificity, with a higher diagnostic performance and confidence as compared with standard CT. Recently, color-coded fat maps derived from abdominal imaging have been proposed for the assessment of bone and soft tissue diseases [21,22]. These reconstructions could further optimize the contrast in the soft tissue around the lumbar spine, due to the presence of fluid in the spinal canal and fat around the nerve roots. In this setting, DECT could allow the detailed visualization of disk abnormalities and possible signs of nerve root involvement; also, DECT could help with the visualization of facet joints or endplates abnormalities or in the detection of disk calcifications and osteophytes [23,24]. DECT could represent a promising alternative to MRI for patients with contraindications [25,26,27]. The aim of this study is to evaluate the accuracy of DECT-based dedicated color-coded fat maps in detecting and grading lumbar disk herniations, using MRI as a reference standard.

## 2. Materials and Methods

Participants: This single-center prospective study was approved by the Institutional Review Board of IRCCS Sacro Cuore Don Calabria Hospital (Prot n. 26650; 26/04/2022; Prog. 3760CESC). Written informed consent was obtained from all participants prior to enrollment. Between September 2024 and May 2025, a total of 43 consecutive patients were considered for inclusion. Patients were referred for second-level radiological imaging by emergency physicians, general practitioners, orthopedic surgeons, and antalgic unit doctors due to lumbar or radiculopathic symptoms. Inclusion criteria were as follows: lumbar or radiculopathic symptoms, availability of DECT and MRI within 3 days, and signed informed consent. Exclusion criteria included a history of lumbosacral surgery and imaging studies with poor image quality.

MRI Protocol: MRI scans were acquired using a 3T scanner (Omega, United Imaging, Shanghai, China) with T1- and T2-weighted sequences and fat suppression in multiplanar acquisition. T1-weighted Fast Spin Echo (FSE) sequences were acquired in the sagittal plane. T2-weighted FSE sequences were acquired in sagittal and axial planes, with fat suppression applied only to sagittal images. Imaging parameters are summarized in Table S1.

DECT Protocol: Non-contrast DECT scans were performed using a dual-source CT scanner (Somatom Definition Force, Siemens Healthineers, Forchheim, Germany). Scanning parameters included tube voltages of 80 and 150 kVp, with a tin filter on the high-energy tube. Tube current was modulated at a 1.6:1 ratio (220 mAs for tube A, 138 mAs reference for tube B). Dose modulation (CARE Dose 4D) ensured radiation levels consistent with previous comparable studies. All patients were scanned in the supine position. Mean volume CT dose index and standard deviation was 8.8 mGy and 3.1 (range, 3.7–12.1 mGy). Mean DLP was 264.2 ± 78.4 mGy·cm (range: 148–398 mGy·cm), with an estimated effective dose of 3.96 ± 1.18 mSv (calculated using k-factor of 0.015 mSv/(mGy·cm) for lumbar spine). All scans employed uniform iterative reconstruction (ADMIRE, strength 3) and automatic dose modulation (CARE Dose 4D) settings across all patients.

Post-Processing: Images obtained at 80 and 150 kV using a soft tissue kernel (Qr32), at 1 mm slice thickness and 0.6 mm interval were transferred to a dedicated workstation (SyngoVia^®^ VB40, Siemens, Erlangen, Germany). Virtual Non-Contrast (VNC) imaging with fat map ‘filter’ was used to generate dedicated reconstructions. The ‘Liver VNC’ preset was selected as it is a vendor default configuration (Siemens Syngo.via VB40) optimized for soft tissue differentiation. The iodine ratio of 1.82 represents the standard factory setting for this preset, ensuring reproducibility across centers using the same platform. For centers using different vendors, equivalent fat–water separation algorithms with similar HU thresholds (−100 to +100 HU for soft tissue) should be employed. These images tend to enhance the contrast between soft tissues, especially between CSF fluid, coded in gray scale, and fat, coded in yellow, whereas intervertebral disks were coded in blue [10].

Image Analysis: Three radiologists with 15, 7, and 5 years of experience in musculoskeletal imaging independently assessed CT, DECT, and MRI datasets. For DECT evaluation, color-coded Liver VNC maps were used with an iodine ratio of 1.82 to optimize discrimination between disk material and adjacent soft tissues. Slice thickness was set, as default, to 1 mm. All readers were free to adjust the slice thickness and the window and the level of super-imposition of dedicated color-coded fat maps. Each disk level was divided into three regions (right, central, left), and a five-score grading was employed for each disk (from 1 to 5: 1 = normal disk; 2 = disk protrusion; 3 = contained herniation; 4 = disk extrusion; 5 = sequestered herniation) [8]. The dichotomization threshold (grades 1–2 vs. 3–5) was selected based on NASS nomenclature recommendations, where grades 1–2 represent disk conditions without true herniation (normal and bulging), while grades 3–5 represent true herniations requiring potential clinical intervention (focal, extruded, and migrated). This grading system was uniformly applied across CT, DECT, and MRI images to enable direct comparison. CT, DECT, and MRI reading sessions were performed randomly, separated by one month delay to reduce the risk of recall bias. No indeterminate results occurred, and all cases were included in the final analysis.

## 3. Statistical Analysis

Statistical analyses were performed to evaluate the diagnostic performance of DECT compared to MRI as the reference standard. Descriptive statistics were calculated for demographic variables, with continuous data expressed as the mean ± standard deviation and categorical variables as frequencies and percentages. The correlation between DECT and MRI grading scores was assessed using Pearson correlation coefficients for each of the three readers, with 95% confidence intervals calculated. The statistical significance was set at *p* < 0.001. To account for the clustering of intervertebral levels within patients, we performed a generalized estimating equation (GEE) analysis with an exchangeable correlation structure. This approach provides robust standard errors that account for the non-independence of observations within patients. The diagnostic concordance between imaging modalities was evaluated by calculating the exact percentage agreement, defined as the proportion of cases where both DECT and MRI assigned identical scores. The chi-square test for homogeneity was used to assess differences in concordance rates among readers (χ^2^ = 1.524, df = 2, *p* > 0.05). Interobserver reliability was measured using Cohen’s Kappa coefficient for all pairwise comparisons between readers, with 95% confidence intervals. Agreement was interpreted according to the Landis and Koch criteria: 0.61–0.80 indicating a substantial agreement and 0.81–1.00 indicating a near-perfect agreement. Additionally, the intraclass correlation coefficient (ICC) was calculated using a two-way random-effects model for absolute agreement to confirm inter-rater reliability. Receiver operating characteristic (ROC) curves were constructed for each reader using the five-point grading scale, with MRI as the reference standard. The area under the curve (AUC) values with 95% confidence intervals were calculated to assess the overall diagnostic performance. For the comparative analysis between conventional CT and DECT, a dichotomous classification was applied where scores of 1–2 were categorized as the absence of herniation, and scores of 3–5 were classified as the presence of herniation. Diagnostic performance metrics including sensitivity, specificity, and accuracy were calculated with 95% confidence intervals for both modalities using MRI as the gold standard. The subgroup analyses were performed, stratifying by age (<50 years vs. ≥50 years) and anatomical location (right-lateral, central, left-lateral), using chi-square tests to identify potential differences in the diagnostic concordance.

## 4. Results

**Figure 1:**
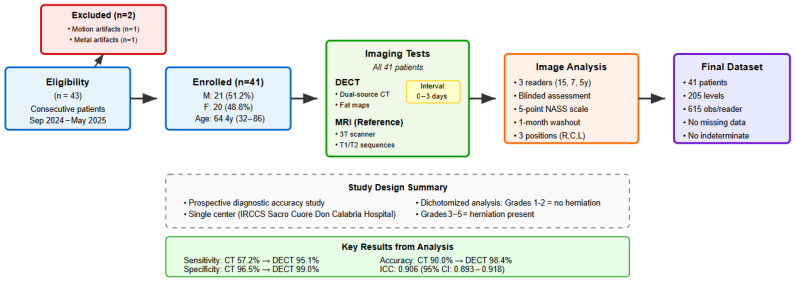
STARD 2015 flow diagram. Forty-three consecutive patients were assessed for eligibility, with two excluded due to imaging artifacts. All 41 enrolled patients underwent both DECT (index test) and MRI (reference standard) within 0–3 days. Image analysis by 3 independent radiologists using 5-point NASS scale resulted in 615 observations per reader (205 levels × 3 positions). No missing data or indeterminate results occurred.

A total of two patients were excluded: one due to motion artifacts on MRI and one due to metal hardware causing artifacts on both MRI and CT (Figure 1).

This study included 41 consecutive patients with lumbar symptoms, for a total of 205 intervertebral levels analyzed (5 levels per patient: L1–L2, L2–L3, L3–L4, L4–L5, L5–S1). The study population had a balanced sex distribution, with 21 male patients (51.2%) and 20 female patients (48.8%). The mean age at the time of examination was 64.4 years, ranging from 32 to 86 years. All patients underwent both dual-energy CT and MRI within 3 days (range 0–3 days, mean 1 day), minimizing the potential temporal bias. Patient data are summarized in Table 1.

**Correlation between Dual-Energy CT and MRI**

**Figure 2:**
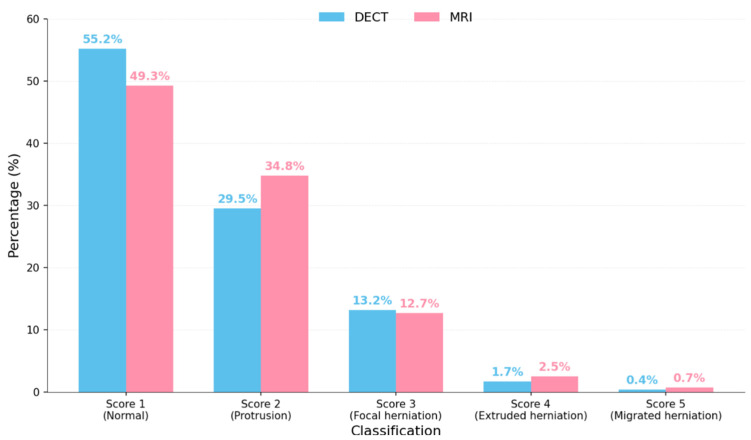
Percentage distribution of classification scores for lumbar disk herniations using dual-energy CT (DECT) and Magnetic Resonance Imaging (MRI). Score 1: normal disk; Score 2: protrusion; Score 3: focal herniation; Score 4: extruded herniation; and Score 5: migrated herniation.

**Figure 3:**
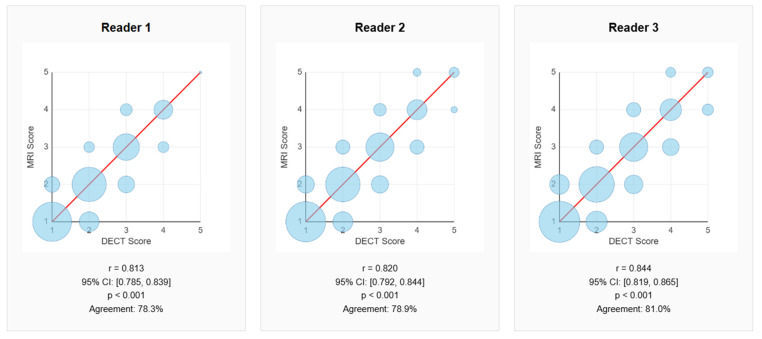
Scatter plots showing the correlation between scores assigned using dual-energy CT (X-axis) and Magnetic Resonance Imaging (Y-axis) by three independent readers. The size of the points is proportional to the frequency of observations. The red dashed line represents a perfect correlation (y = x). All readers show a strong and statistically significant correlation (*p* < 0.001) with Pearson correlation coefficients greater than 0.81.

The correlation analysis between the scores assigned to DECT and MRI images showed a strong positive association across all three readers. (Table 2, mean Pearson’s r = 0.826, *p* < 0.001) The Pearson correlation coefficient was 0.813 (95% CI: 0.785–0.839) for reader 1, 0.820 (95% CI: 0.792–0.844) for reader 2, and 0.844 (95% CI: 0.819–0.865) for reader 3, with a mean correlation of 0.826. All coefficients were statistically significant (*p* < 0.001), with t-values ranging from 34.5 to 38.9 (Figure 2 and Figure 3). These results indicate a significant agreement between the two imaging modalities in the assessment of lumbar disk herniations. The GEE analysis confirmed these findings, with adjusted *p*-values remaining as <0.001.

**Inter-rater Reliability**

The intraclass correlation coefficient (ICC) for the absolute agreement was 0.906 (95% CI: 0.893–0.918), confirming excellent inter-rater reliability according to established criteria.

**ROC Analysis**

**Figure 4:**
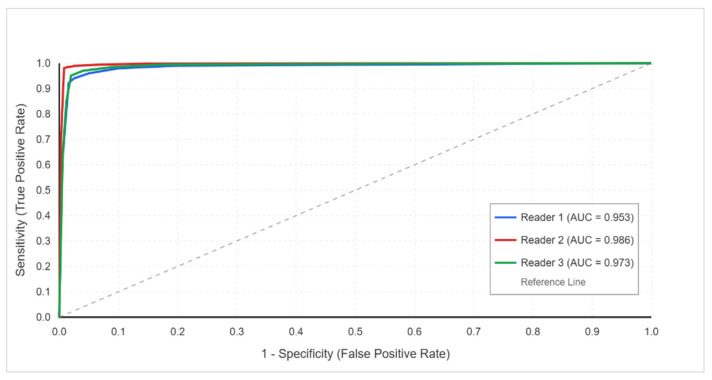
Receiver operating characteristic (ROC) curves for DECT diagnostic performance using MRI as the reference standard. All three readers demonstrated excellent discrimination ability, with AUC values exceeding 0.95 (reader 1: AUC = 0.953, 95% CI: 0.924–0.982; reader 2: AUC = 0.986, 95% CI: 0.970–1.002; reader 3: AUC = 0.973, 95% CI: 0.950–0.995). The curves show consistently high sensitivity and specificity across all diagnostic thresholds, confirming DECT’s robust performance in detecting lumbar disk herniations.

The ROC curve analysis demonstrated an excellent diagnostic performance across all readers: reader 1 AUC = 0.953 (95% CI: 0.924–0.982), reader 2 AUC = 0.986 (95% CI: 0.970–1.002), and reader 3 AUC = 0.973 (95% CI: 0.950–0.995). These values indicate the outstanding discrimination ability of DECT in differentiating herniated from non-herniated disks (Figure 4).

**Figure 5:**
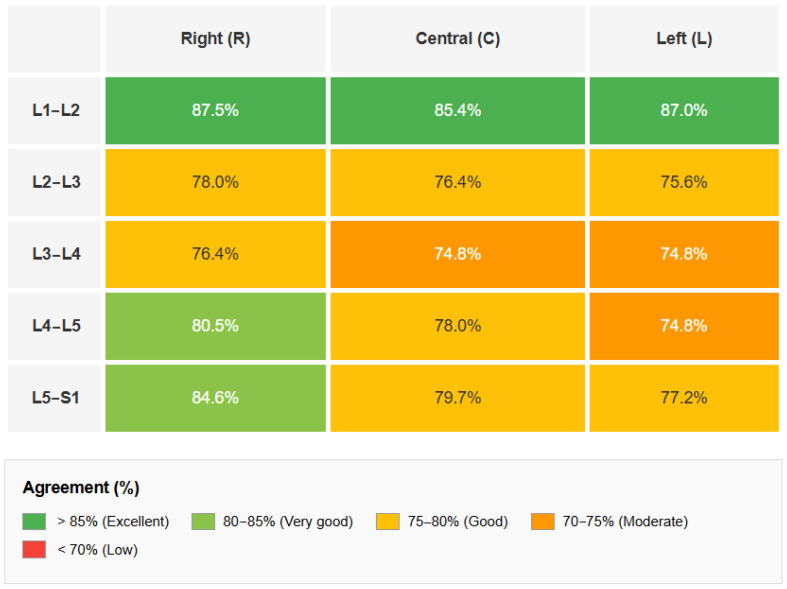
Percent agreement between dual-energy CT and Magnetic Resonance Imaging stratified by intervertebral level (L1–L2 to L5–S1) and anatomical position (right, central, left).

**Diagnostic Concordance Analysis:** The exact percentage agreement between DECT and MRI—defined as the proportion of cases in which both modalities are assigned the same score—was 78.3% (95% CI: 74.9–81.4%) for reader 1, 78.9% (95% CI: 75.5–81.9%) for reader 2, and 81.0% (95% CI: 77.7–83.9%) for reader 3, with an overall average of 79.4%. The chi-square test for homogeneity among readers did not show significant differences (χ^2^ = 1.524, df = 2, *p* > 0.05), confirming the consistency of evaluations regardless of the observer. The analysis by the intervertebral level (Table 3 and Figure 5) revealed the highest agreement at L1–L2 (86.7%), followed by L5–S1 (80.4%) and L4–L5 (77.8%). Levels with relatively lower agreements were L3–L4 (75.3%) and L2–L3 (76.7%), although all levels demonstrated a concordance above 75%.

**Score Distribution and Pathological Patterns:** The analysis of the score distribution revealed that 52.2% of overall evaluations (DECT + MRI) corresponded to normal disks (score 1), 32.1% corresponded to disk protrusions (score 2), 13.0% corresponded to focal herniations (score 3), 2.1% corresponded to extruded herniations (score 4), and only 0.5% corresponded to migrated herniations (score 5). When analyzed separately, DECT showed a slight tendency to underestimate the pathology compared to MRI, with 55.2% of score 1 assignments versus 49.3% for MRI, although this difference was not statistically significant.

**Figure 6:**
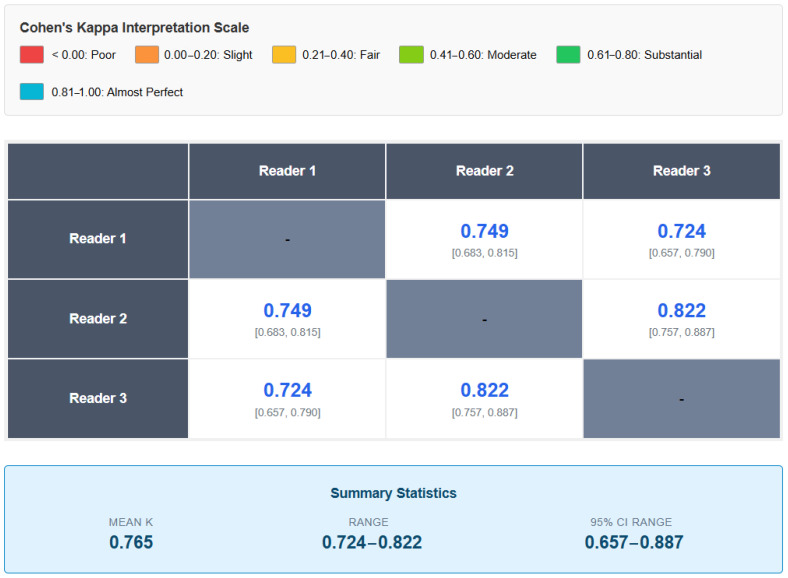
Symmetric matrix showing Cohen’s Kappa coefficient values for inter-reader agreement in DECT assessments. The diagonal cells are left blank as they represent self-comparisons. All pairwise comparisons demonstrate substantial agreement (κ > 0.72), with the agreement between readers 2 and 3 reaching near-perfect levels (κ = 0.822). The 95% confidence intervals are reported below each Kappa value. All pairwise comparisons show substantial agreement (κ > 0.70), with the comparison between readers 2 and 3 reaching almost perfect agreement (κ = 0.822). The mean Cohen’s Kappa of 0.765 indicates substantial overall interobserver reliability for DECT assessments.

**Interobserver Agreement:** The analysis of the interobserver agreement using Cohen’s Kappa coefficient showed substantial values for all pairs of readers. The Kappa between readers 1 and 2 was 0.749 (95% CI: 0.683–0.815), between readers 1 and 3 was 0.724 (95% CI: 0.657–0.790), and between readers 2 and 3 was 0.822 (95% CI: 0.757–0.887). The mean Kappa value (0.765) indicates a substantial agreement according to the Landis and Koch criteria (Table 4 and Figure 6), with the comparison between readers 2 and 3 reaching a near-perfect agreement (κ > 0.80).

**Subgroup Analysis:** The stratified analysis by age included 7 patients (17.1%) under 50 years and 34 patients (82.9%) aged 50 or older. The DECT–MRI agreement was 76.2% in the younger group and 80.1% in the older group, with no statistically significant differences (χ^2^ = 2.381, *p* > 0.05). The analysis by anatomical location showed a slight variability in concordance: right-lateral (n = 68, agreement 81.3%, *p* = 0.42), central (n = 69, agreement 79.0%, *p* = 0.38), and left-lateral (n = 68, agreement 77.9%, *p* = 0.31). The chi-square test for homogeneity showed no significant differences among locations (χ^2^ = 1.89, *p* = 0.39).

**Comparison between Conventional CT and DECT in the Diagnosis of Disk Herniations:** To complete the analysis and assess DECT’s ability to identify all herniations (not only clinically severe ones), a further dichotomous analysis was performed with a different diagnostic threshold: scores of 1–2 were classified as an absence of herniation (0), and scores of 3–5 were classified as a presence of herniation (1), using MRI as the reference standard (Table 5).

**Figure 7:**
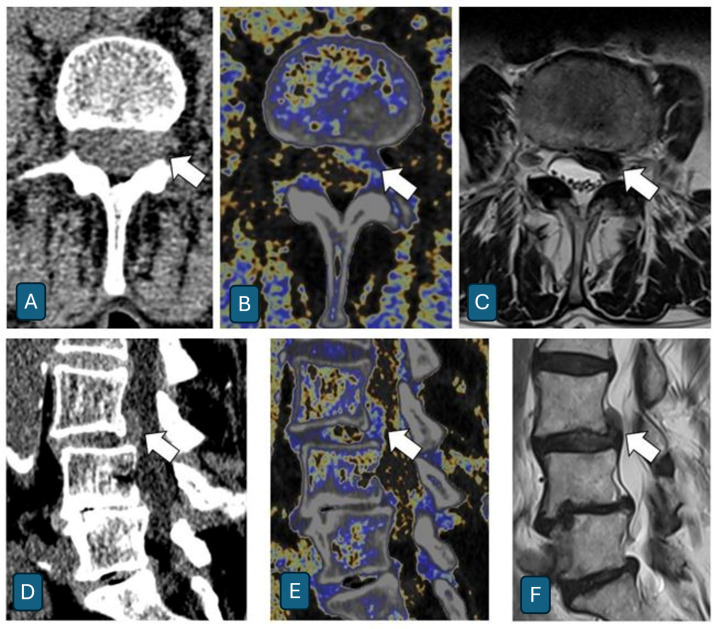
A 55-year-old male patient with a history of left-sided lumbar radiculopathy/lumbalgia. Axial and sagittal imaging demonstrates a posterolateral left-sided extruded disk component with partial cranial migration beneath the posterior longitudinal ligament (white arrow). Compared to conventional CT images (**A**,**D**), DECT images (**B**,**E**) enhance the contrast differentiation between discal structures (in blue) and cerebrospinal fluid (in grayscale), allowing an optimal diagnostic assessment. MRI T2 images (**C**,**F**) represent the current gold standard.

**Figure 8:**
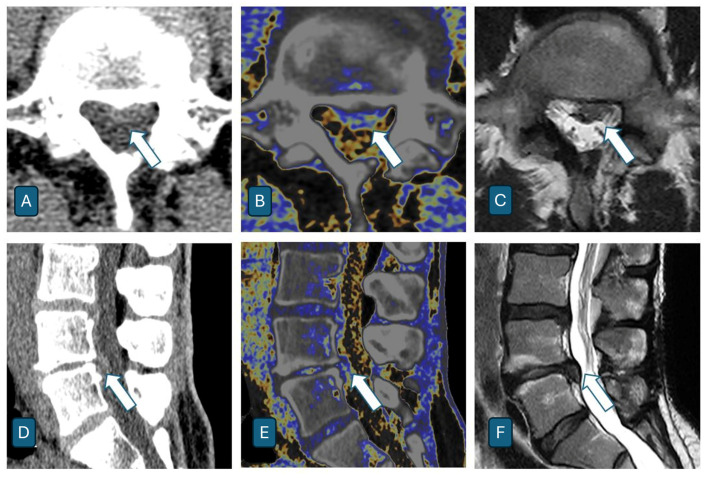
A 48-year-old female patient presenting with chronic midline lumbar pain. Axial and sagittal imaging reveal a predominantly central lumbar focal disk herniation with a small posterolateral left-sided component (white arrow). DECT images (**B**,**E**) provide a superior tissue contrast by employing a color-coded map that distinctly separates the herniated disk material (blue) from the surrounding bone and cerebrospinal fluid (displayed in grayscale). This colorimetric enhancement makes it easier to identify the herniation margins and differentiate them from adjacent structures, which is more challenging on conventional CT (**A**,**D**). Although MRI images (**C**,**F**) remain the gold standard for soft tissue evaluation, DECT represents a valuable imaging option, particularly when MRI is contraindicated or unavailable.

The analysis demonstrated a marked superiority of DECT over conventional CT. The mean sensitivity was 95.1% for DECT versus 57.2% for conventional CT, showing an increase of 37.9%. Specificity remained high for both modalities (DECT: 99.0%; CT: 96.5%), while the overall diagnostic accuracy improved from 90.0% to 98.4%. The most significant improvement was observed in the reduction in false negatives. While conventional CT failed to correctly identify approximately half of the herniations (sensitivity between 51.0% and 62.7% among readers), DECT demonstrated a markedly superior detection capability (sensitivity 92.2–98.0%), while maintaining excellent specificity. Representative cases are shown in Figure 7 and Figure 8. Detailed 2 × 2 contingency tables for all readers are provided in Supplementary Table S2.

## 5. Discussion

In this prospective study, the diagnostic performance of DECT with dedicated color-coded fat map reconstructions in detecting and grading lumbar disk herniations was evaluated, using MRI as the reference standard. The results demonstrate a strong correlation between DECT and MRI assessments (mean Pearson’s r = 0.826), with high levels of diagnostic concordance (average exact agreement: 79.4%) and substantial interobserver agreement (Cohen’s κ = 0.765). These findings confirm the potential of DECT to serve as a reliable alternative imaging modality in the evaluation of lumbar disk pathologies. The method proved to be reliable regardless of the patient age or intervertebral level examined, with a particularly high performance at L1–L2 and L5–S1 levels. Also, there were only few differences in the diagnostic performance among readers, so the experience of radiologist should play a limited role in the assessment of disk abnormalities. The results aligned with previous studies that have highlighted the advantages of spectral CT in musculoskeletal imaging. Notably, Booz et al. [20] reported comparable sensitivity and specificity for DECT in lumbar disk herniation assessments using virtual noncalcium maps, although this study further supports these findings with a larger number of intervertebral levels analyzed and the integration of fat map reconstructions, which enhanced the soft tissue contrast. In this study, color-coded reconstructions significantly improved the visualization of the disk morphology, enabling a better delineation of protrusions, extrusions, and sequestered fragments. As expected, fat maps allowed for an enhanced contrast between the cerebrospinal fluid, epidural fat, and disk material. This contributed to the high reader agreement, especially among the two most experienced radiologists (κ = 0.822), indicating that with adequate training regarding the use of post-processing tools, DECT interpretations can be highly reproducible. Furthermore, DECT demonstrated the greatest concordance at the L1–L2 and L5–S1 levels, which are often challenging for standard CT due to anatomical variations and overlapping structures. The consistency of DECT across all lumbar levels, with a concordance >75%, supports its utility across a range of spinal morphologies. The relatively lower agreement at L3–L4 (75.3%) and L2–L3 (76.7%) levels, though still substantial, may be attributed to several anatomical and technical factors. These mid-lumbar levels experience the greatest biomechanical stress during flexion–extension movements, potentially leading to more subtle and dynamic disk changes that are challenging to characterize on static imaging. Additionally, the transitional zone between the lordotic curve and the more mobile lower lumbar segments at these levels may create partial volume effects on axial reconstructions. The presence of a more prominent epidural venous plexus at these levels could also potentially affect the color-coded fat map reconstructions, as the vascular structures may create subtle artifacts that influence the disk boundary delineation. Despite these challenges, the agreement remained above 75%, supporting the robustness of the DECT technique across all lumbar levels. The analysis confirmed the superiority of DECT over conventional CT in diagnosing disk herniation. In particular, the mean sensitivity significantly increased for DECT versus conventional CT, whereas the specificity remained high for both modalities. These results confirm that the addition of spectral information and dedicated color maps provides DECT with a substantial diagnostic advantage over conventional CT, bringing its performance closer to that of MRI in the evaluation of lumbar disk herniations. Despite the overall strong performance of DECT, some limitations must be acknowledged. A small subset of cases (particularly at the L3–L4 and L2–L3 levels) showed discrepancies between DECT and MRI scores. These discrepancies primarily involved the underestimation of the pathology by DECT, likely due to mild protrusions or early degenerative changes that were not sufficiently highlighted by current reconstruction parameters. Moreover, while DECT offers an excellent tissue contrast for CT, it cannot fully replicate the soft tissue resolution and neural detail provided by MRI, particularly for evaluating subtle nerve root compression, perineural inflammation, or non-calcified epidural lesions. Additionally, although temporal bias was minimized by performing both exams within 3 days, transient disk hydration changes could have potentially influenced the image interpretation. Another limitation is the potential operator dependence of the post-processing and color map interpretation, especially in less experienced readers. This underscores the need for standardized protocols and automated segmentation tools to ensure consistency in clinical practice. The findings of this study suggest that DECT, when combined with optimized color-coded reconstructions, may serve as a valuable alternative for evaluating lumbar disk herniations, especially in scenarios where MRI is contraindicated (e.g., claustrophobia, implanted devices) or unavailable. Its rapid acquisition time, broader availability, and growing integration in routine workflows position DECT as a viable tool in both acute and preoperative settings. Moreover, DECT’s ability to detect disk calcifications, osteophytes, and bone marrow edema adds complementary information not always captured by MRI, thus enhancing the overall diagnostic yield, particularly in complex or postoperative cases. Future research should aim to further refine DECT protocols and reconstruction algorithms, potentially incorporating AI-driven segmentation to enhance diagnostic accuracy and reduce observer variability. Comparative cost-effectiveness analyses and patient outcome studies are also warranted to evaluate the clinical impact of adopting DECT in routine spine imaging. Larger multicenter studies with diverse populations would help validate these results and extend generalizability. Finally, the integration of DECT with other functional imaging biomarkers (e.g., perfusion or quantitative bone density) may provide a more comprehensive assessment of the lumbar spine pathology.

## 6. Conclusions

This study confirms that dual-energy CT with dedicated color-coded fat map reconstructions demonstrates a high diagnostic concordance with MRI in evaluating lumbar disk herniations. DECT shows excellent sensitivity (94.6%), specificity (98.8%), and interobserver agreement (κ = 0.765), making it a promising alternative in cases where MRI is not feasible. With continued technical refinements and broader adoption, DECT may play an increasingly central role in the diagnostic algorithm of lumbar spine disorders.

## Figures and Tables

**Table 1:** Demographic and clinical characteristics of the study population.

Number of Patients	41
Age (years)	
- Mean ± DS	64.4 ± 13.2
- Range	32–86
Sex, n (%)	
- Male	21 (51.2%)
- Female	20 (48.8%)
Intervertebral Levels Examined	
- Total Levels	205
- Levels Per Patient	5
- Levels Distribution	All examined
Examined Levels	L1–L2, L2–L3, L3–L4, L4–L5, L5–S1

**Table 2:** Correlation and agreement between DECT and MRI by reader.

Reader	Pearson Correlation (r)	IC 95%	*p*-Value	Agreement (%)	IC 95% Agreement
1	0.813	0.785–0.839	<0.001	78.3	74.9–81.4
2	0.820	0.792–0.844	<0.001	78.9	75.5–81.9
3	0.844	0.819–0.865	<0.001	81.0	77.7–83.9
Average	0.826	--	--	79.4	--

**Table 3:** DECT–MRI agreement by intervertebral level.

Level	Average Agreement (%)	Reader 1 (%)	Reader 2 (%)	Reader 3 (%)
L1–L2	86.7	87.0	84.6	88.6
L2–L3	76.7	74.8	77.2	78.0
L3–L4	75.3	75.6	75.6	74.8
L4–L5	77.8	74.8	78.0	80.5
L5–S1	80.4	79.5	78.9	82.9

**Table 4:** Interobserver agreement (Cohen’s Kappa) for DECT assessments.

Comparison	Kappa	IC 95%	Interpretation
Reader 1 vs. 2	0.749	0.683–0.815	Substantial
Reader 1 vs. 3	0.724	0.657–0.790	Substantial
Reader 2 vs. 3	0.822	0.757–0.887	Almost Perfect
Average	0.765	--	Substantial

**Table 5:** Comparison of diagnostic performance between conventional CT and DECT (with 95% confidence intervals).

Parameter	CT	DECT	Delta (Δ)
Reader 1			
Sensitivity (%)	51.0 (41.4–60.5)	92.2 (85.3–96.0)	+41.2
Specificity (%)	96.1 (94.1–97.5)	98.4 (97.0–99.2)	+2.3
Accuracy (%)	88.6 (85.9–90.9)	97.4 (95.8–98.4)	+8.8
Reader 2			
Sensitivity (%)	57.8 (48.1–67.0)	98.0 (93.1–99.5)	+40.2
Specificity (%)	96.3 (94.3–97.6)	99.2 (98.0–99.7)	+2.9
Accuracy (%)	89.9 (87.3–92.1)	99.0 (97.9–99.6)	+9.1
Reader 3			
Sensitivity (%)	62.7 (53.1–71.5)	95.1 (89.0–97.9)	+32.4
Specificity (%)	97.1 (95.2–98.2)	99.4 (98.3–99.8)	+2.3
Accuracy (%)	91.4 (88.9–93.3)	98.7 (97.5–99.3)	+7.3
Average			
Sensitivity (%)	57.2	95.1	+37.9
Specificity (%)	96.5	99.0	+2.5
Accuracy (%)	90.0	98.4	+8.4

## Data Availability

Data are available on specific request.

## Supplementary Materials

The following supporting information can be downloaded at: https://www.mdpi.com/article/10.3390/jcm14197000/s1, Table S1: MRI Sequence Parameters; Table S2: 2×2 Contingency Tables—All Readers Combined.
