# Supplementary material for: Assessment of Intervertebral Lumbar Disk Herniation: Accuracy of Dual-Energy CT Compared to MRI
Source: J Clin Med. 2025 Oct 3;14(19):7000. doi: 10.3390/jcm14197000 (PMC12524339; doi:10.3390/jcm14197000)
Supplement: Supplementary file 1 [file jcm-14-07000-s001.zip › jcm-3862427-supplementary.pdf]

Table S1. MRI Sequence Parameters

Parameter	T1 FSE Sagittal	T2 FSE Sagittal	T2 FSE Axial
**Repetition Time (TR)**	550 ms	3500 ms	4000 ms
**Echo Time (TE)**	10 ms	100 ms	110 ms
**Field of View (FOV)**	280 × 280 mm	280 × 280 mm	200 × 200 mm
**Matrix Size**	320 × 256	384 × 307	320 × 256
**Slice Thickness**	4 mm	4 mm	3 mm
**Slice Gap**	0.4 mm	0.4 mm	0.3 mm
**Fat Suppression**	No	Yes	No

Table S2. 2×2 Contingency Tables - All Readers Combined.

Reader	2×2 Table	Performance Metrics							
	**Test Result**	**MRI+**	**MRI-**	**Total**	**Sensitivity (95% CI)**	**Specificity (95% CI)**	**Accuracy (95% CI)**	**PPV**	**NPV**
**Reader 1**	**CT+**	52	20	72	51.0% (41.4-60.5)	96.1% (94.1-97.5)	88.6% (85.9-90.9)	72.2%	90.8%
	**CT-**	50	493	543					
	**Total**	102	513	615					
**Reader 2**	**CT+**	59	19	78	57.8% (48.1-67.0)	96.3% (94.3-97.6)	89.9% (87.3-92.1)	75.6%	92.0%
	**CT-**	43	494	537					
	**Total**	102	513	615					
**Reader 3**	**CT+**	64	15	79	62.7% (53.1-71.5)	97.1% (95.2-98.2)	91.4% (88.9-93.3)	81.0%	92.9%
	**CT-**	38	497	535					

	**Total**	102	512	614*					
**Average**	**57.2%**	**96.5%**	**90.0%**		**76.3%**	**91.9%**			

A. Conventional CT vs MRI (Reference Standard)

**Reader**	**2×2 Table**	**Performance Metrics**							
	**Test Result**	**MRI+**	**MRI-**	**Total**	**Sensitivity (95% CI)**	**Specificity (95% CI)**	**Accuracy (95% CI)**	**PPV**	**NPV**
**Reader 1**	**DECT+**	94	8	102	92.2% (85.3-96.0)	98.4% (97.0-99.2)	97.4% (95.8-98.4)	92.2%	98.4%
	**DECT-**	8	505	513					
	**Total**	102	513	615					
**Reader 2**	**DECT+**	100	4	104	98.0% (93.1-99.5)	99.2% (98.0-99.7)	99.0% (97.9-99.6)	96.2%	99.6%
	**DECT-**	2	509	511					
	**Total**	102	513	615					
**Reader 3**	**DECT+**	97	3	100	95.1% (89.0-97.9)	99.4% (98.3-99.8)	98.7% (97.5-99.3)	97.0%	99.0%
	**DECT-**	5	510	515					

	**Total**	102	513	615					
**Averag e**	**95.1%**	**99.0%**	**98.4%**		**95.1%**	**99.0%**			

B. DECT vs MRI (Reference Standard)

Reader	Δ Sensitivity	Δ Specificity	Δ Accuracy	Δ PPV	Δ NPV
**Reader 1**	+41.2%	+2.3%	+8.8%	+20.0%	+7.6%
**Reader 2**	+40.2%	+2.9%	+9.1%	+20.6%	+7.6%
**Reader 3**	+32.4%	+2.3%	+7.3%	+16.0%	+6.1%
**Average**	**+37.9%**	**+2.5%**	**+8.4%**	**+18.8%**	**+7.1%**

C. Diagnostic Improvement (DECT vs CT)

*One observation excluded in Reader 3 CT analysis due to technical reasons
CI = Confidence Interval; PPV = Positive Predictive Value; NPV = Negative Predictive Value
MRI+ = MRI Positive; MRI- = MRI Negative
Dichotomization: Grades 1-2 = negative (no herniation); Grades 3-5 = positive (herniation present)
